# Pulmonary inflammatory myofibroblastic tumor in a male child: A case report

**DOI:** 10.1002/ccr3.6003

**Published:** 2022-06-21

**Authors:** Seyed‐Javad Seyedi, Amin Saeidinia, Parisa Dehghanian

**Affiliations:** ^1^ Pediatric Department, Faculty of Medicine Mashhad University of Medical Sciences Mashhad Iran; ^2^ Pharmaceutical Research Center Mashhad University of Medical Sciences Mashhad Iran; ^3^ Pathology Department, Akbar Hospital Mashhad University of Medical Sciences Mashhad Iran

**Keywords:** inflammatory myofibroblastic tumor, pediatric population, pulmonary tumor

## Abstract

Pulmonary inflammatory myofibroblastic tumor (IMT) is a rare condition in the normal population and specifically in the pediatric population. We reported a 9‐year‐old male child who presented with cough and intermittent fever and weight loss that was most suggestive of the infectious process. We reviewed the consideration of diagnosis and treatment.

## Abstract 2

Pulmonary IMT is a rare pulmonary mass that might be asymptomatic or manifest by hemoptysis, cough, constitutional symptoms, and pneumonia. It should be in the mind that IMT might mimic neoplasms, by history, radiology, and clinical aspects.

## 1 INTRODUCTION

The occurrence of primary pulmonary neoplasms in pediatric patients is uncommon. Inflammatory myofibroblastic tumor (IMT) is a rare neoplasm, which has a moderate risk of malignancy appearing mainly in children and young adults.^1^, ^2^ Its nature is in the majority of cases, benign and rarely can metastases however could commonly relapse.^3^ This tumor is composed of several inflammatory cells, and the diversity of which ranges from mainly myofibroblastic cells to plasma cells in the pathological examination.^4^

Inflammatory myofibroblastic tumor is defined as “a lesion composed of a proliferation of Myo‐fibroblastic spindle and stellate cells with abundant eosinophilic cytoplasm mixed with infiltrative plasma, inflammatory cells, lymphocytes, and eosinophils” in the World Health Organization (WHO).^5^ IMT is known by numerous terms, including plasma cell granulomas, inflammatory pseudo‐tumor, fibrous histiocytoma, and pseudo‐lymphoma,^6^ and may occur in a wide range of anatomical locations such as the lungs, omentum, bladder, spleen, breast, pancreas, liver, colon, spermatic cord, prostate, peripheral nerves, soft tissue, and orbit. About one‐third of these tumors are found in the respiratory tract.^6^ We here report a case of IMT of the lung.

## 2 CASE PRESENTATION

**FIGURE 1:**
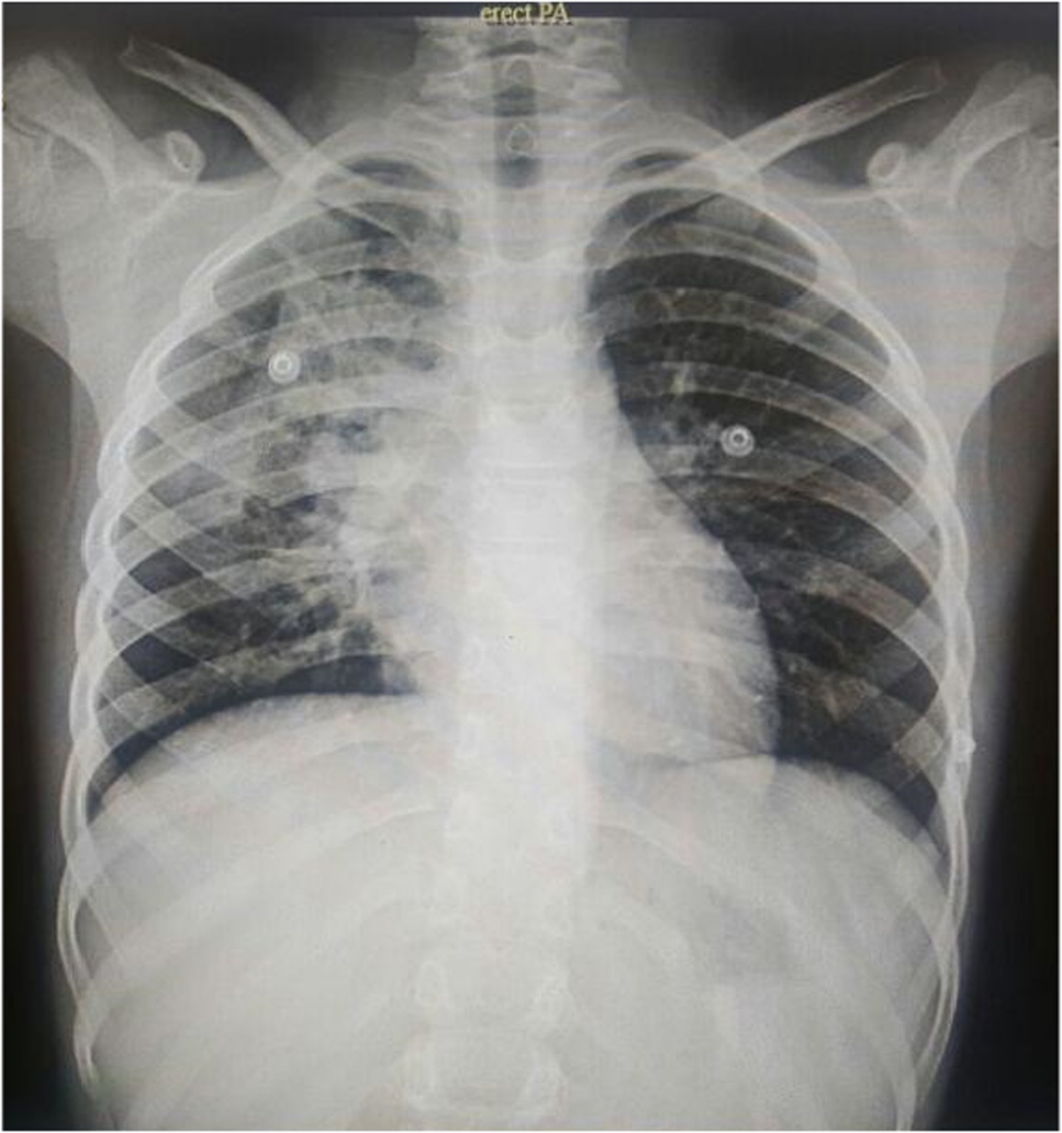
Chest X‐Ray

A 9‐year‐old male child with chronic productive cough and occasional fever since 6 months ago was referred to our clinic. He had severe weight loss in this period. Two months before admission, he had massive hemoptysis. He had a history of tonsillectomy 2 years ago and no exposure to tuberculosis case. There was no history of the familial pulmonary disorder. At admission, his vital signs were stable (HR: 88/min, BP: 100/80 mmHg, RR: 18/min, and T: 37.2°C). In the examination, there was decreased pulmonary sound in the right lung. He had no positive drug or social history. Exposure to irritant substances or smoke was not reported with no history of allergy. There was clubbing in the upper and lower limbs' fingers. There was no shortness of breath or respiratory distress. There was no pathologic issue in his chest and abdominal sonography. His first laboratory data showed a leukocytosis (WBC 22,300 with PMN dominant pattern [85%]). ESR and CRP were in the nearly normal range. Anti‐Echinococus antibody was negative. Other laboratory data were in normal range. His chest X‐ray is shown in Figure 1. In his pre‐admission computer tomography (CT) scan, a mass‐like foci in the right lung was reported.

**FIGURE 2:**
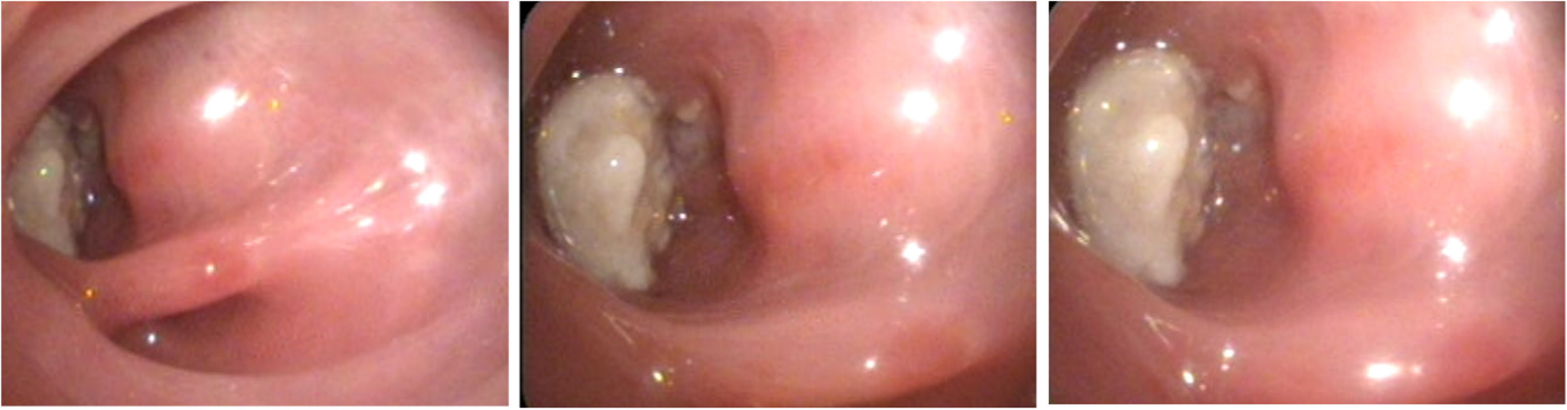
Bronchoscopy; the upper division of right bronchus that is obstructed by a mass

He was admitted to our center for more evaluation. After admission, antibiotic therapy and supportive care were performed. He underwent bronchoscopy. In fibro‐optic bronchoscopy, upper division of the right bronchus was completely obstructed. After washing, various cystic membranes were seen in the airway. The mucosa was erythematous and inflamed with dense secretions that were suctioned (Figure 2).

After the surgical consult, he was a candidate for surgery and underwent right upper lobectomy and wedge resection. After thoracotomy, a mass‐like lesion with granulomatous tissue was resected. There was a fistula that was closed by resection. Chest tube size 28 was inserted, and the sample was sent for pathologic examination.

**FIGURE 3:**
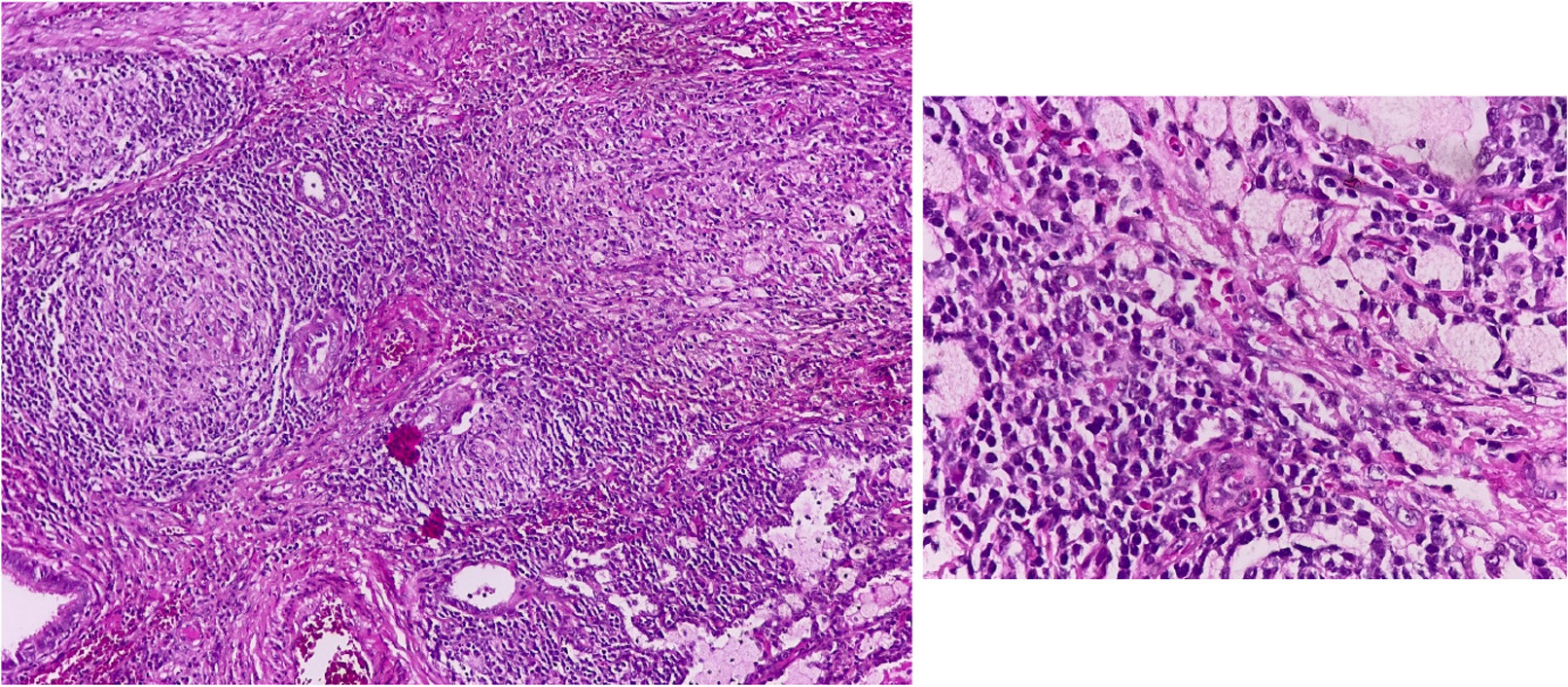
Histopathological H&E staining showing inflammatory cells

The pathologist assessed a wedge biopsy of the lung, with the brown outer surface, measuring 2.5 × 2 × 1 cm, and a central creamy‐yellow nodular lesion, measuring 1 cm in diameter. Sections of the lung tissue with pleural fibrous thickening reported, that is revealing prominent interstitial nodular lymphoid proliferations surrounding large epithelioid cells (resembling granulomas), and few of them with are large nucleolated cells without necrosis. A diffused intra‐alveolar infiltration of foamy histiocytes was noted. In addition, a central focus of pulmonary necrosis with mixed acute inflammatory cell infiltration (abscess formation) was present. Foci of hemorrhage were also reported. In Zhiel–Neelson staining, acid‐fast bacilli were not seen. PPD test was negative. Because of the nodular lymphoid proliferation of pulmonary interstitium with one necrotic area and abscess formation, it was suggestive of interstitial lymphocytic pneumonia or granulomatous disease and we ruled out it by checking CD20, CD3, CD15, and CD30 that have shown no definite evidence of Hodgkin lymphoma. Inflammatory myofibroblastic tumor (IMT) was diagnosed by its pattern (Figure 3). The patient was discharged after the surgery and is follow‐up now for probable relapse. The length of follow‐up was 3 years.

## 3 DISCUSSION

Inflammatory myofibroblastic tumor is one of the rare low‐to‐intermediate grade sarcomas. Initially, it was thought to be an inflammatory response to various stimuli, but recent studies have proved IMT to be neoplastic and can recur locally and metastasize.^7^ It has also been suggested that trauma, surgery, autoimmune etiologies, inflammation, and infections such as Epstein–Barr virus or human herpes virus could result in the development of IMT.^8^

Inflammatory myofibroblastic tumor was first described in the lungs but later was also found on other sites such as the orbit, spleen, genitourinary tract, mesentery, cardioesophageal junction, breast, central nervous system, and larynx. The larynx has been a very rare site for involvement in IMT.^9^ Children with IMT may exhibit symptoms of chronic inflammation as a low‐grade fever, weight loss, anemia, thrombocytosis, polyclonal hyper‐gammaglobulinemia, and elevated sedimentation rate. Several cases are asymptomatic and are detected only incidentally in imaging studies. Among patients with endobronchial lesions, symptoms of bronchial irritation such as cough and hemoptysis may be accompanied by chest pain.^10^

In our case, chest X‐ray, history of weight loss, and the occurrence of intermittent fever and hemoptysis, besides endemic status, made us suspicious of infectious processes like tuberculosis or echinococcosis. Because of the negative staining of the BAL sample and laboratory data, we thought about the non‐infectious process like malignancies because of weight loss. Despite doing bronchoscopy and HRCT, the diagnosis was last after histopathological assessment. According to previous studies, there are only 26 published cases of pediatric pulmonary IMT (the age between 3 and 13 years), even though the real incidence is presumed to be higher.^3^, ^11^ Peripheral lung lesions appear to be more frequent than central and endobronchial tumors that may be present about in 10% of the cases resulting in bronchial obstruction and atelectasis.^12^

Inflammatory myofibroblastic tumor can be sometimes diagnosed as an incidental finding on a routine CXR.^13^ In all previous cases, at the time of presentation, patients had a fever, respiratory distress, arthralgia, clubbing, night sweat, vomiting, and hemoptysis, and at the onset, fever and cough were the commonest symptoms.^3^ There has been an ongoing controversy about whether an IMT is a reactive lesion or a true neoplasm.^14^ Although its incidence is fairly scarce, the existing literature clearly defines its relative similarities in terms of clinico‐pathological and radiological findings and almost uniformly favors surgical resection as a mainstay for the most efficient management strategy. The recurrence rate remains low, and a 10‐year survival rate is around 80%.^15^

Treatment is primarily a complete, but conservative surgical excision. This approach is necessary to prevent recurrence.^16^, ^17^ An appropriate histologic assessment should be obtained before the surgery (needle biopsy by bronchoscopy), in order to avoid an unnecessary procedure.^17^

## 4 CONCLUSION

Pulmonary IMT is a rare disorder with significant complications among the child population. It may be non‐specific in presenting in this population and patients undergo different antibiotic treatments before definite diagnosis. While radiological techniques can help in diagnosis, confirmation of the diagnosis should be performed by histopathological assessment. The choice of treatment is based on complete surgical resection.

## AUTHOR CONTRIBUTION

Dr. Seyed‐Javad Seyedi, Dr. Amin Saeidinia, and Dr. Parisa Dehghanian were responsible for the case treatment, development of methodology, writing of the manuscript, and confirming the final version.

## CONFLICTS OF INTERESTS

There is no conflict of interest.

## CONSENT

The authors have confirmed that patients' consent has been signed and collected in accordance with the journal's patient consent policy.

## Data Availability

The data that support the findings of this study are available from the corresponding author upon reasonable request.
