#  Corrigendum

**DOI:** 10.1002/ccr3.6051

**Published:** 2022-07-14

In the article entitled “Pulmonary inflammatory myofibroblastic tumor in a male child: A case report,” which was previously published in Volume 10 Issue 6 of Clinical Case Reports, the last author's name was misspelt. The correct name should be Paria Dehghanian.
